# Autumn Is Coming

**DOI:** 10.31486/toj.24.5044

**Published:** 2024

**Authors:** Ronald G. Amedee

**Affiliations:** Director of Clinical School and Professor, The University of Queensland Medical School, Ochsner Clinical School, New Orleans, LA; Editor-in-Chief, *Ochsner Journal*

*And all at once, summer collapsed into fall….*

*–Oscar Wilde*

In the Fall 2024 issue of *Ochsner Journal*, four original research articles, one literature review, and seven case reports and clinical observations serve as the principal elements for the edition. We also have a letter to the editor—and the authors’ reply—commenting on a case report published in this issue about an atypical form of pneumonia found in patients being treated for solid organ malignancy. An editorial authored by one of Ochsner's own anesthesiologists, Bobby Nossaman, entitled “So What?” is an interesting commentary on the statistical analyses of clinical studies results. Finally, the issue contains the seventh installment of the quarterly Health, Medicine, and Society column, this time authored by Rachel S. Dauterive, Ochsner Department of Bariatric Surgery, who details “The Cautious Promise of GLP-1 Receptor Agonists,” drugs initially approved for treatment of patients with type 2 diabetes mellitus but now commonly used to treat obesity.

In “Simple and Epididymal-Sparing Orchiectomy for Surgical Castration in Stage IV Prostate Cancer,” the first original research article in the issue, Louisiana State University (LSU) clinicians show the efficacy of orchiectomy as an alternative to medical androgen deprivation therapy for patients diagnosed with metastatic prostate cancer. For “Characterization of High-Risk-Other Human Papillomavirus Genotypes in Papanicolaou Tests, High-Grade Squamous Intraepithelial Lesions, and Cervical Cancer,” researchers at LSU and Woman's Hospital in Baton Rouge, Louisiana, teamed up on an analysis of human papillomavirus (HPV) genotypes in Pap test samples and biopsy tissues, discovering that the three most common HPV genotypes they identified in the HPV Pap test samples are not covered by the 9-valent HPV vaccine. Providers at Hennepin Healthcare in Minneapolis, Minnesota, and the University of Rochester, New York, present their findings from the retrospective study, “Endoscopic Management of Pancreaticobiliary Injuries: A Level 1 US Trauma Center Experience.” Ochsner anesthesiologists, led by Bobby Nossaman, analyzed the “Risk of Instrumental Delivery in Maternal Obesity: Estimates With Measures of Effect Size.” In their analysis, risk differences with 95% confidence intervals were used as measures of effect size to assess the magnitude and precision of previously reported comorbidities on the need for instrumental delivery in obese parturients. They found that maternal obesity did not play a role in the need for instrumental delivery.

The literature review in this issue addresses “Beta-Blocker Usage in Patients With Heart Failure With Reduced Ejection Fraction During Acute Decompensated Heart Failure Hospitalizations.” Authors Brennan, Harmouch, Basit, and Alraies accompany their review with a guideline-based flowchart to help physicians make informed decisions regarding beta-blocker therapy.

Case reports and clinical observations include “Ectopic Pancreatic Tissue in the Gallbladder” by Kaur, Garg, and Sharma, followed by “Reverse Total Shoulder Arthroplasty in an Adult With Achondroplasia” by Ochsner orthopedists Suri, Grilliot, Renshaw, Jones, and colleagues. The remarkable case of a young woman from Ukraine is presented in “Recurrent Intracranial Ewing Sarcoma” submitted by clinicians from Berlin, Germany. Mishra, Mateen, Jabeen, et al discuss “Sheehan Syndrome Unmasked by Adrenal Crisis Secondary to Severe Dengue Fever,” and Jackson, Isern, Jesina, et al present diagnostic and treatment details for two patients in their case series “*Pneumocystis jirovecii* Pneumonia in Patients Treated for Solid Organ Malignancy.” “Catecholamine-Induced Cardiomyopathy Secondary to Pheochromocytoma Rescued With Percutaneous Left Ventricular Assist Device: Novel Application of the Impella CP in a Pediatric Patient” is authored by another Ochsner team of Zumwalt, Roybal, Crittendon, Lucas, and Wells. Finally, a report from Balani, Malage, et al discusses a “Rare Presentation of Leiomyoma of the Pancreas.”

The autumnal equinox, occurring on Sunday, September 22, 2024, marks the arrival of fall. New Orleanians will not experience a sudden *collapse into fall*, as we expect to have hot and humid days well after September 22, but the equinox does mean that less humid and cooler days are on the way. Football season arrives, Halloween signals the end of October, and our clocks fall back in early November (accompanied by an extra hour of sleep).

Ochsner continues to celebrate excellence with the naming of Dr Salima Qamruddin to the inaugural Edward D. Frohlich, MD Endowed Mentorship Position which not only honors the legacy of Dr Frohlich but also aims to further the mentorship and development of future leaders in cardiovascular care. Dr Qamruddin's impressive background and her commitment to excellence make her an ideal recipient of this endowed chair. The Edward D. Frohlich, MD Endowed Mentorship Position is the second endowed professorship at Ochsner. The first was the John Ochsner Heart and Vascular Institute Distinguished Professor in Cardiovascular Innovation, a position currently held by Dr Hernan Bazan. Congratulations to Salima and Hernan on these prestigious achievements.

